# The knowledge of the increased risk of complications in multiple pregnancies does not affect the desire to transfer more than one embryo in *in vitro* fertilisation treatment

**DOI:** 10.5935/1518-0557.20140021

**Published:** 2014

**Authors:** Edson Borges Jr, Amanda S. Setti, Daniela P.A.F. Braga, Rose Marie Melamed, Rita Figueira, Assumpto Iaconelli Jr.

**Affiliations:** 1 Fertility - São Paulo/SP, Brazil

**Keywords:** FMD, endothelial function, brachial artery, menstrual cycle

## Abstract

**Objetive:**

One of the main complications in in vitro fertilisation (IVF) is multiple pregnancies. This study was designed to investigate how many embryos subjects participating in an online survey would want to transfer in their IVF cycles.

**Methods:**

This study was conducted in a Brazilian private assisted fertilisation centre. Individuals who accessed the centre’s website were asked to participate in the survey. The survey was based on important information concerning multiple gestations, followed by a single multiple choice question, as follows: ‘Knowing that the transfer of one embryo reduces the chance of pregnancy, and that the transfer of more than one embryo could result in multiple pregnancies, which comes with risks to the mother and the babies, answer: how many embryos would you transfer in your IVF cycle?’. There were three available answers: one, two or three embryos.

**Results:**

A total of 1,049 subjects participated in the survey: 109 males and 940 females. The majority of the participants answered that they would like to have two embryos transferred (53.7%); followed by three embryos (35.0%), and one embryo (11.3%).

**Conclusion:**

Men and women tend to underestimate the risks of complications associated with multiple embryo transfers and multiple gestations. It is the physician’s responsibility to consider single embryo transfer (SET) as the method of choice and perform double or triple embryo transfers only in special circumstances.

## INTRODUCTION

Over the past few decades the use of assisted reproductive technologies (ART) has significantly increased and made gestation possible for many infertile couples. ART are initiated with a controlled ovarian stimulation, which results in the retrieval of many oocytes, leading to the transfer of multiple embryos in order to maximise pregnancy rates. As a consequence, ART has been associated with a 30-fold increase in multiple pregnancies, when compared to spontaneous pregnancies (ACOG, 2005).

Multiple pregnancies are associated with many negative consequences for the mother and the foetuses. The odds of pregnancy-induced hypertension, pre-eclampsia, gestational diabetes, postpartum hemorrhage and postpartum depression are the most frequent complications for the mother (Ontario, 2006). Babies from multiple pregnancies are at significantly higher risk of early death, premature birth, low birth weight, and mental and physical disabilities related to prematurity (Ontario, 2006).

Increasing awareness of maternal and foetal complications has led to a reduction in the number of embryos transferred in in vitro fertilisation clinics, but the frequency of twin pregnancies is still high (Maheshwari *et al.*, 2011). A reduction in the number of embryos transferred might, however, contradict the patient’s desire to achieve a successful outcome and thereby increase the necessity for further IVF attempts.

Several studies have investigated patient preferences for embryo transfer policy and for outcomes of IVF treatment (Gleicher *et al.*, 1995, Grobman *et al.*, 2001, Kalra *et al.*, 2003, Pinborg *et al.*, 2003, Child *et al.*, 2004, Murray *et al.*, 2004, Ryan *et al.*, 2004, Blennborn *et al.*, 2005, Hojgaard *et al.*, 2007, Newton *et al.*, 2007, Ryan *et al.*, 2007, Twisk *et al.*, 2007, Hope and Rombauts, 2010, Fiddelers *et al.*, 2011, Newton *et al.*, 2013). The majority of studies focusing on patient attitudes have shown that patients generally have a preference for double over SET (Murray *et al.*, 2004, Hojgaard *et al.*, 2007, Newton *et al.*, 2007, Ryan *et al.*, 2007, Twisk *et al.*, 2007). In addition, patients tend to prefer twins over a single baby (Gleicher *et al.*, 1995, Grobman *et al.*, 2001, Pinborg *et al.*, 2003, Hojgaard *et al.*, 2007).

The provision of relevant information about the risks has been reported to decrease the preference for multiple embryo transfers among IVF patients (Newton *et al.*, 2007, Hope and Rombauts, 2010). As patients are actively involved in clinical decision-making, understanding patient desires and knowledge about multiple gestations is important. This study was designed to investigate how many embryos would subjects participating in an online survey want to transfer in their IVF cycle, after being informed about the risks associated with multiple embryo transfer.

## MATERIAL AND METHODS

**Figure 1:**
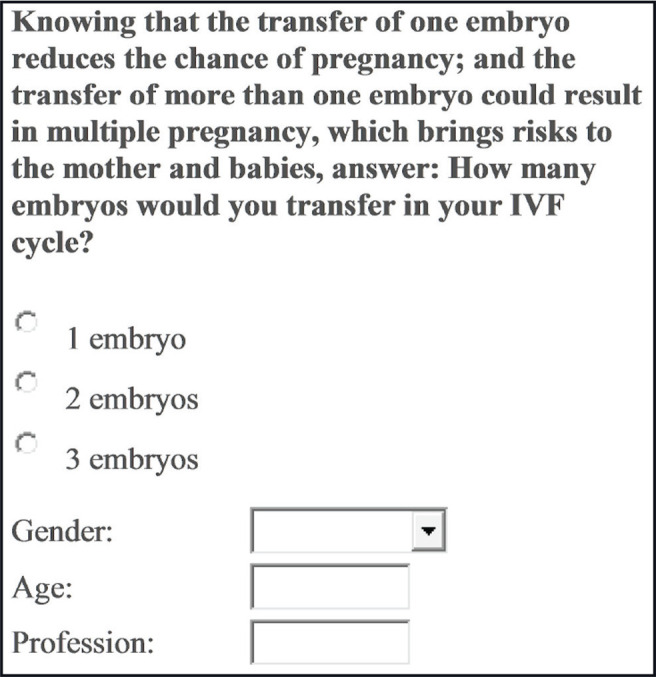
Illustration of the survey

This study was conducted in a Brazilian private assisted fertilisation centre. Individuals who accessed the centre’s website from September 2013 to May 2014 were asked to participate in the survey. The survey provided two important pieces of evidence, followed by a single multiple choice question, as follows: ‘Knowing that the transfer of one embryo reduces the chance of pregnancy, and that the transfer of more than one embryo could result in multiple pregnancies, which brings risks to the mother and babies, answer: how many embryos would you transfer in your IVF cycle?’. There were three available answers: (i) one embryo, (ii) two embryos, and (iii) three embryos. In addition, participants were asked to enter their age, gender and profession (Figure 1).

Data are expressed as mean ± standard deviation. Data analysis was conducted using MINITAB 16 Software.

## RESULTS

**Figure 2:**
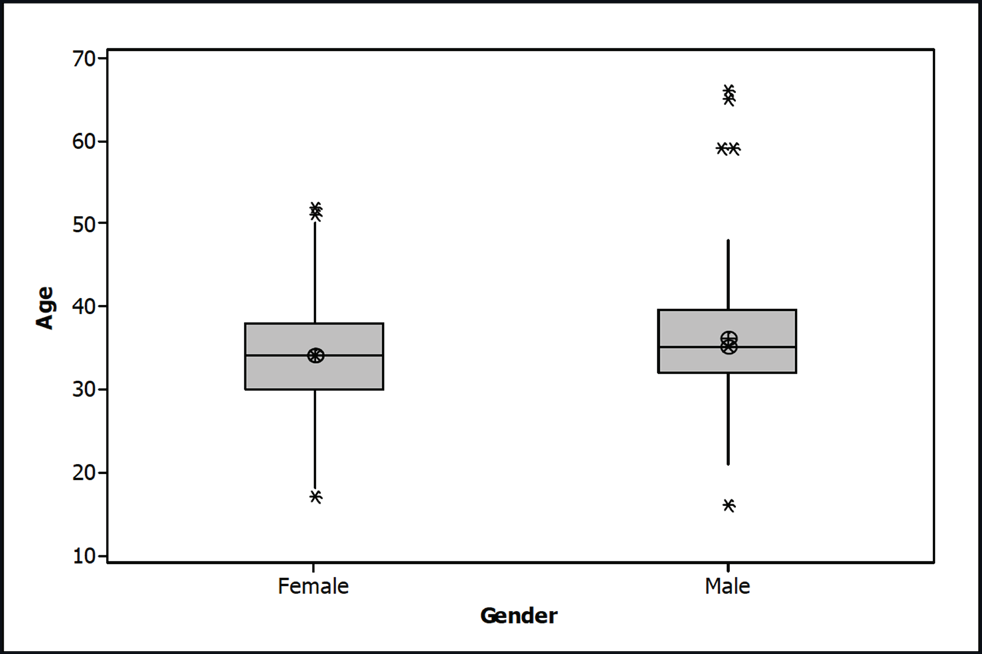
Box plot of female and male participants’ age

**Figure 3:**
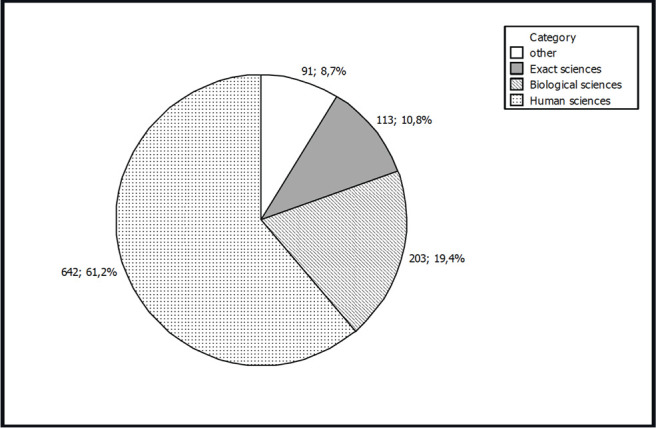
Participants’ professional áreas

**Figure 4:**
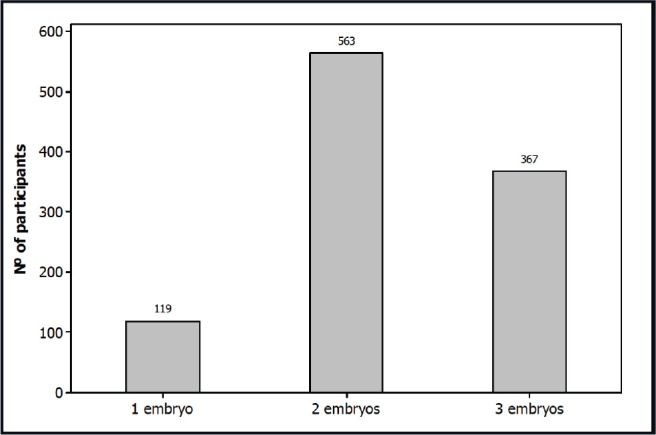
Result of survey

**Figure 5:**
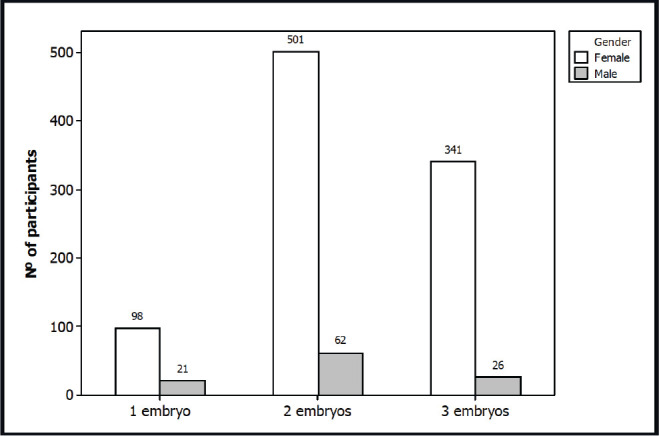
Result of survey by gender

A total of 1,049 subjects participated in the survey: 109 males (10.4%) and 940 females (89.6%). The mean age was 34.2 ± 5.1 years. The mean female age was 35.7 ± 5.8 (range: 17-52) and the mean male age was 36.0 ± 8.2 years (range: 16-66) (Figure 2). Regarding professions, a total of 642 (61.2%) participants were related to human sciences, 203 (19.3%) to biological sciences, 113 (10.8%) to exact sciences and 91 (8.7%) were unemployed, retired or students (Figure 3). The result of the survey is shown in Figures 4 and 5. The majority of the participants answered that they would like to have two embryos transferred (n=563; female=501, male=62; 53.7%); followed by three embryos (n=367; female=341, male=26; 35.0%), and one embryo (n=119, female=98, male=21; 11.3%).

## DISCUSSION

The aim of this survey was to investigate how many embryos the subjects participating in an online survey would want to transfer in their IVF cycle. Despite the provision of risk information regarding multiple pregnancies, the majority of subjects participating in this survey preferred to have two embryos transferred, followed by three embryos and one embryo, respectively.

Several survey-based studies have investigated patient opinion regarding embryo transfer policy and pregnancy outcomes. Our findings corroborate those from similar studies, suggesting that patients generally have a preference for double over SET (Murray *et al.*, 2004, Hojgaard *et al.*, 2007, Newton *et al.*, 2007, Ryan *et al.*, 2007, Twisk *et al.*, 2007). The embryo transfer decision seems to be influenced by an understanding of the risks associated with multiple pregnancies, estimates of the likelihood of single and multiple pregnancy, and demographic factors (Newton *et al.*, 2007, Van Peperstraten *et al.*, 2008). There is also evidence that emotional state can influence decision-making and the process of risk assessment (Fiddelers *et al.*, 2011, Newton *et al.*, 2013). Moreover, it has been proposed that the supposed risk of not achieving a pregnancy might supersede medical concerns and, therefore, the patient makes what seems to them to be a safer decision to transfer more embryos (Newton *et al.*, 2007; Newton *et al.*, 2013).

In addition, patients do not always share physician concerns regarding multiple pregnancies, and show some resistance to SET (Ryan *et al.*, 2004). Gleicher *et al.* (1995) found that fear of multiple pregnancies was rejected by 64% of surveyed patients and that up to 90% of couples desired the conception of twins. Primary reasons for preferring twins are desire for siblings, a positive attitude towards twins, a wish to minimise physical and psychological stress through having as few IVF treatments as possible (Hojgaard *et al.*, 2007), nulliparity, lower family income, younger patient age, prior evaluation for infertility, longer duration of infertility, and lack of knowledge regarding risks of multiple gestations (Ryan *et al.*, 2004).

In Brazil, a recent resolution from the Federal Medicine Council (CFM) on IVF treatments defined that: (i) for patients under the age of 35, no more than two embryos should be transferred; (ii) for patients between 36-39 years of age, no more than three embryos should be transferred; (iii) for patients between 40-50 years of age, no more than four embryos should be transferred. However, these recommendations do not take into consideration the patients’ prognosis and/or previous failed IVF cycles, nor the stage at which the embryo is transferred; cleavage-stage or blastocyst (CFM 2013).

Meanwhile, in the United States of America, the following guidelines, provided by the American Society for Reproductive Medicine and the Society for Assisted Reproductive Technology are recommended: (i) patients under the age of 35 with a favourable prognosis should be offered a SET and no more than two embryos (cleavage-stage or blastocyst) should be transferred; (ii) for patients between 35-37 years of age with a favourable prognosis, no more than two cleavage-stage embryos should be transferred. All others in this age group should have no more than three cleavage-stage embryos or two blastocysts transferred; (iii) for patients between 38-40 years of age with a favourable prognosis, no more than three cleavage stage embryos or two blastocysts should be transferred. All others in this age group should not have more than four cleavage-stage embryos or three blastocysts transferred; (iv) for patients between 41-42 years of age, no more than five cleavage-stage embryos or three blastocysts should be transferred. In every age group cited above, for patients with two or more previously failed fresh IVF cycles or a less favourable prognosis, one additional embryo may be transferred according to individual circumstances. There are insufficient data to recommend a limit on the number of embryos to transfer in women over 43 years of age (Practice Committee of American Society for Reproductive and Practice Committee of Society for Assisted Reproductive, 2013).

Most societies have issued guidelines to aid the decrease in the number of embryos transferred in IVF cycles and, consequently, the risk of multiple pregnancies. Although there is sufficient evidence demonstrating that elective SET may eliminate multiple pregnancies without compromising the cumulative live birth rate, many physicians are reluctant to adopt SET. In fact, in a previous study performed by our group, the ART professionals’ attitudes towards their own IVF cycles were investigated (Bonetti *et al.*, 2008). It was observed that the transfer of a higher number of embryos and the associated multiple pregnancy risks were seen as acceptable, illustrating that these professionals have similar attitudes and perceptions to those in the lay population and the participants of the present survey.

It is important to emphasise that a survey of this kind is subject to certain methodological limitations.

The results may have been influenced by patient self-selection because (a) not everyone is connected; (b) even if connected, not all potential participants are equally computer literate; (c) a participant might have answered the questionnaire twice or more; (d) the vast majority of participants are female; (e) the fertility potential of the participants is unknown, and (f) participants were not informed of the pregnancy rates when one, two or three embryos are transferred.

The conclusion of this study is that men and women are not sufficiently aware of, or tend to underestimate the risks of complications associated with multiple embryo transfers and multiple gestations. The provision of risk information did not influence the acceptance of SET. The physician has to give necessary and qualified information to the couple to help them with the decision-making process regarding the number of embryos to be transferred. Nonetheless, it is the physician’s responsibility to consider SET as the method of choice and perform double or triple embryo transfers only in special circumstances.
